# Real-Time Monitoring of Thermal Phenomena during Femtosecond Ablation of Bone Tissue for Process Control

**DOI:** 10.3390/bioengineering11040309

**Published:** 2024-03-26

**Authors:** Samy Al-Bourgol, Guillaume Machinet, Aboubakr Bakkali, Marc Faucon, Laura Gemini

**Affiliations:** ALPhANOV, Institut d’Optique d’Aquitaine, 33400 Talence, France; samy.albourgol@gmail.com (S.A.-B.); marc.faucon@alphanov.com (M.F.)

**Keywords:** femtosecond laser, bone tissue ablation, bone tissue thermodynamics, laser-induced breakdown spectroscopy

## Abstract

Femtosecond (fs) laser technology is currently being considered in innovative fields such as osteotomy and treatment of hard tissue thanks to the achievable high resolution and ability to prevent tissue damage. In a previous study, suitable process parameters were obtained to achieve competitive ablation rates on pork femur processing. Nevertheless, a better control of thermal accumulation in the tissue during laser ablation could further improve the postoperative regeneration of the treated bone compared with conventional procedures and push forward the exploitation of such technology. This study presents methods for real time analyses of bone tissue temperature and composition during fs laser ablation and highlights the importance of implementing an efficient cooling method of bone tissue in order to achieve optimized results. Results show that it is possible to achieve a larger process window for bone tissue ablation where bone tissue temperature remains within the protein denaturation temperature in water-based processing environment. This is a key outcome towards a clinical exploitation of the presented technology, where higher process throughputs are necessary. The effects of process parameters and environments on bone tissue were confirmed by LIBS technique, which proved to be an efficient method by which to record real-time variation of bone tissue composition during laser irradiation.

## 1. Introduction

Osteotomy is a routine procedure in which a bone is cut to correct a defect, its orientation or appearance. Although the use of lasers in osteotomy has been extensively studied in recent years, commercially available laser systems for surgical applications have been implemented just very recently and in only a limited number of occasions [1,2]; consequently, the cutting of hard tissue is still dominated by the use of mechanical methods through the use of saws, drills and burrs [3]. Mechanical instruments are very effective in achieving high ablation rates but cause a lot of thermal and physical damage to the bone tissue, preventing its postoperative regeneration and inducing detrimental and often dangerous fragmentation into numerous small bone chips [4].

The use of the laser technology allows for the removal of bone tissue in a contactless way, thus greatly reducing tissue damage, and can be easily transported by optical fibers allowing high precision positioning and accuracy, as well as the possibility to ablate complex 3D shapes [5,6,7,8,9,10,11]. Currently, laser systems employed in the clinical environment, for applications including enamel ablation in dentistry, skin ablation in dermatology and bone surgery, are mainly quasi-continuous wave (QCW) or continuous wave (CW). The Er-YAG laser is of great importance in the medical field because of its emission wavelength (2.94 μm) which corresponds with strong absorption peaks of both water and hydroxyapatite, key components of hard biological tissue [12]. Indeed, hydroxyapatite and water together represent up to 80% of the total composition of a human bone [13]. Nonetheless, QCW, CW lasers are also linked to important irradiation-induced carbonization, calcination and tissue damage due to the important related thermal loads. Slightly reduced but still important detrimental thermal effects were observed when a pulsed laser source with pulse duration of about 200 ns and wavelength of 532 nm was employed on porcine mandible samples, while no thermal damage was visible upon irradiation with 200 fs and 775 nm laser light on the same sample [14], demonstrating the key role of the laser pulse duration in the interaction between the laser and the hard tissue.

Fs lasers are consequently gaining a special place in several biomedical applications for which high precision and highly localized interventions are required [15,16,17] due to their special ability to minimize thermal effects, as demonstrated by their current clinical use in fields such as ophthalmology. The current drawback of these laser sources, limiting their wider exploitation in the surgical environment, lies in a low ablation efficiency, far lower than that achievable by mechanical tools [18,19]. In a previous work, a laser process parameter window was identified through which to achieve the highest ablation rate on pork femur without thermal damage to the bone tissue with industrially available fs laser sources [20]. Nevertheless, in order to further improve this value by employing high average power fs laser sources, thermal dynamics during the laser irradiation of bone tissue needs to be addressed and controlled. 

The temperature must be carefully monitored during laser treatment to ensure the survival of biological tissue. Indeed, proteins are the building blocks of all biological tissues. These proteins each have unique structural and functional characteristics that guarantee the proper operation of an organ. The protein loses its function if this structure is denatured as a result of exposure to high temperatures, which can cause issues with how the related biological tissue functions or regenerates. Although cortical and cancellous bone decontamination above 50 °C has not been shown to cause any significant thermal damage [21], an average temperature above 41 °C might already lead to protein denaturation [22,23]. At temperatures above 60 °C, the collagen filaments are denatured. In addition, these filaments decompose at temperatures above 100 °C, and carbonize at temperatures above 150 °C [24,25]. Beyond 400 °C, changes in the crystallography of the hydroxyapatite are visible [26] leading to burning of the tissue over a temperature of about 1000 °C [27].

The way the laser pulse energy is delivered and absorbed by the bone tissue is the key parameter to monitor in order to control the temperature and the thermal damage of biological materials. To obtain an efficient and quasi-athermal ablation process, as in the femtosecond regime of interaction, a localized amount of energy is transferred to a highly-absorbing material in a period of time which is shorter than the time required for the material excited electrons to reach a thermal equilibrium with the surrounding lattice [28]. From this point, the material’s characteristic thermal diffusivity, defined as the rate at which heat is transferred from a high temperature zone to a lower temperature one, i.e., the rapidity at which the material reaches its initial temperature, has to be considered. When two successive laser pulses impinge on the same area at a faster rate than its thermal diffusivity, thermal accumulation occurs [29,30]. Carbonization and calcination of the bone are the consequences of a thermal relaxation of the tissue that is slower than the thermal accumulation induced by the laser [28].

In this work, different methods were employed to monitor, analyze and finally control the heat dynamics of bone tissue upon industrial fs laser processing at two different irradiation wavelengths (1030 nm and 515 nm) and varying laser processing parameters. The thermal accumulation on the irradiated bone tissue was investigated by varying the spatial overlap between successive laser pulses. Different water-based and air-based processing environments were evaluated to observe the influence of a cooling system on the bone tissue temperature. The temperature of the bone tissue during laser processing was quantified by thermal imaging (FLIR camera) and thermocouples measurements. Finally, laser-induced breakdown spectroscopy (LIBS) was employed as a real-time diagnostic system with which to observe the variation of the bone tissue elemental composition during laser processing in relation to the temperature analyses [31,32]. Post-processing characterization methods were carried out by scanning electron microscopy (SEM) and energy dispersive X-ray (EDX) analysis to qualitatively observe laser-induced morphological changes and the degree of elemental modification induced in the bone tissue after laser processing. Results demonstrate the possibility of maintaining the temperature of the bone tissue within acceptable values during the whole laser process by using water-based cooling methods coupled with moderate spatial overlap between successive laser pulses, without strongly affecting the ablation rates.

## 2. Materials and Methods

### 2.1. Preparation of Biological Samples of Porcine Femur

Porcine femurs were collected from the same butcher and labelled with the age and sex of the animal. The femurs were conserved at a temperature of −6 °C. Before treatment, they were left to defrost for about 1 h at room temperature in a laminar air-flow protection system (Thermo Scientific HERAsafe KS12, Courtaboeuf Cedex, France) and cleaned with a scalpel in order to remove the marrow and all soft tissue. Remains of fat were removed by a pre-cleaning step in a solution of deionized (DI) water and ethanol 70%. The samples were then left drying at room temperature for about 1 h. Finally, the femurs were mechanically cut by a diamond blade in order to obtain smaller size samples (approx. 1 × 1 cm^2^) to facilitate handling during all processing and characterization steps. All samples employed for the laser processing were obtained from the diaphysis section of the femurs. Table 1 summarize the procedural steps implemented for all tests.

### 2.2. Experimental Setup

Two different laser sources from Amplitude (Pessac, France) were employed for the tests: (i) a Satsuma HP3 laser system (central wavelength of 1030 nm, maximum average power 40 W, maximum repetition rate 1 MHz) for the LIBS analyses only, and (ii) a Tangerine laser system (central wavelength of 1030 nm, maximum average power 20 W, maximum repetition rate 1 MHz). For the ablation tests, the latter was coupled to an optical module able to convert the fundamental wavelength to its second harmonic (515 nm), allowing it to work in visible regime. The pulse duration was in the order of 350 fs for both laser sources. A detailed description and a schematic representation of the experimental setups employed for the tests can be found in [20].

**Figure 1:**
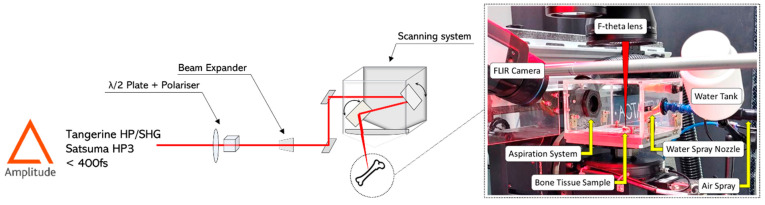
Scheme of the experimental setup employed for all tests.

For all tests, areas with a dimension of 3 × 4 mm^2^ were laser processed on the bone samples with a scanning geometry that was composed by overlapping lines with interline distance *h*, each line being composed from overlapping laser pulses of focused spot diameter *Φ* and inter-pulse distance *d*. The distance *d* between two successive pulses is given by the ratio between the velocity *v* at which the scan head deflects the beam on the sample surface and the laser repetition rate *RR*: *d* = *v*/*RR*. Thermal effects were investigated by varying the scanning speed *v* and the interline distance *h* so that a percentage of spatial overlap between neighbor pulses could be defined as horizontal overlap (*HOL* = (*Φ* − *d*)/*Φ*) and vertical overlap (*VOL* = (*Φ* − *h*)/*Φ*), respectively. Finally, in order to obtain ablation cavities characterized by measurable ablation depths within the limit of the lens focal depth, a number N of 5 successive passes was selected for all tests. Table 1 summarizes the window of process parameters employed for the ablation tests. A scheme of the experimental setup employed for the tests is presented in Figure 1. 

### 2.3. Protocols for Real-Time Characterization

Four different cooling methods were employed to control the bone tissue temperature during laser ablation:

- A control environment without any active cooling system (labelled “Without cooling”).
- An air-jet cooling system with controlled air pressure (labelled “Air-knife”) placed 10 cm from the bone surface at a 45-degree angle of incidence.
- A water-based system with complete immersion of the bone tissue (labelled “Water immersion”) under a 300 µm thin layer of DI water at room temperature (20 °C).
- A hybrid air-and-water jet system which sprayed high-pressure water on to the processed tissue (labelled “Water spray”) every 1 s. Each water pulse sent to the sample corresponded to a quantity of 200 µL of DI water. Between each water pulse, air was sprayed onto the sample until the next water pulse at 4 bar.

**Figure 2:**
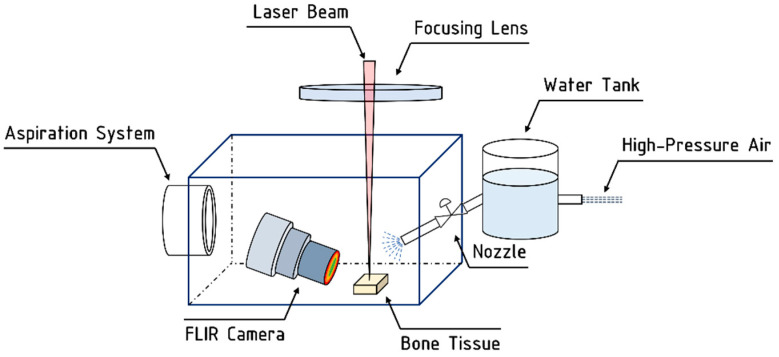
Schematic representation of the customized setup employed for laser processing in hybrid air-and-water jet system.

In order to avoid contamination of the f-theta lens used to focus the laser beam onto the bone surface, a customized protection cage equipped with an aspiration system was built in order to safely process the sample. The system is pictured in Figure 2. 

### 2.4. Processing Environment for Bone Tissue Cooling

For a complete and comprehensive characterization of the bone tissue behavior during fs laser processing, two different real-time inline monitoring systems were employed simultaneously: thermal imaging by IR thermal camera to measure the temperature of the bone tissue and LIBS analyses to track its elemental composition changes. These two systems were calibrated and tested before laser processing to verify the reliability and solidity of the collected data.

#### 2.4.1. Thermal Imaging

An IR thermal camera (Teledyne FLIR, model A655, Wilsonville, OR, USA) placed above the sample at an angle of 45 degrees was employed for temperature measurements during the ablation tests. The videos were acquired at 50 fps using the ResearchIR software (Teledyne FLIR, Wilsonville, OR, USA). A pre-acquisition calibration was performed to calibrate the emissivity index of the samples which was set to 0.95 [28]. Videos were recorded for each test in two measurement ranges: from −40 °C to 150 °C and from 150 °C to 600 °C. In order to verify the reliability of the measurements with respect to the angle of view between the camera and the sample surface, measurements at four different viewing angles from 20° to 90° were carried over a hot plate containing 5 bone samples kept at 60 °C. Before the measurements, the bone samples were heated on the plate for 1 h until a stable sample temperature was reached. 

**Figure 3:**
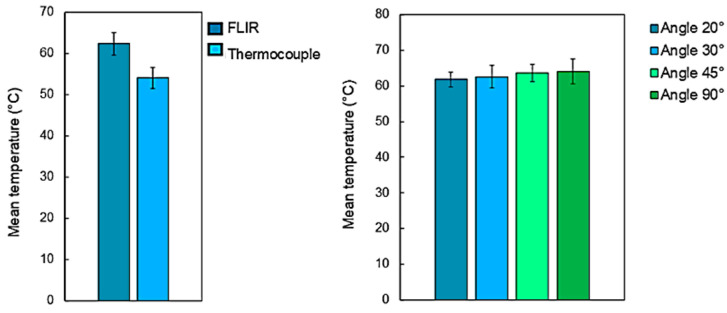
(**left-hand side**) Evolution of the mean temperature of the bone tissue measurement by thermal camera and thermocouples and (**right-hand side**) influence of the camera’s viewing angle on the acquired temperatures. Bone tissue samples were place on a hot plate kept at a temperature of 60 °C.

The results obtained by thermal imaging were then compared with the results obtained with a thermocouple K (USB TC-08, Pico Technology, Eaton Socon, UK) on the same samples. For each view angle, five acquisitions were undertaken by thermocouple on five different zones of the same sample. This approach allowed for the determination of a mean temperature for each sample at each angle of view. Figure 3 presents the mean temperature registered on the five different samples acquired by thermocouple measurements and thermal imaging at four different angles of view of the FLIR camera. 

Regardless of the angle of view of the camera in relation to the sample surface, the temperature measurements showed no important differences, with variations within <5.4% of the average values. Important variations were also not observed between temperature measurements acquired with the thermal camera and the thermocouple with variations within <4.6% of the average values. These results confirm the accuracy of temperature measurements acquired throughout all laser processing tests. To identify a meaningful parameter which could define a representative temperature during each different laser process carried at different processing parameters, a mean temperature is here introduced and defined as the average maximal temperature recorded on the bone tissue surface at each laser passage and on each sample treated with the same set of laser process parameters.

#### 2.4.2. LIBS Analyses 

**Figure 4:**
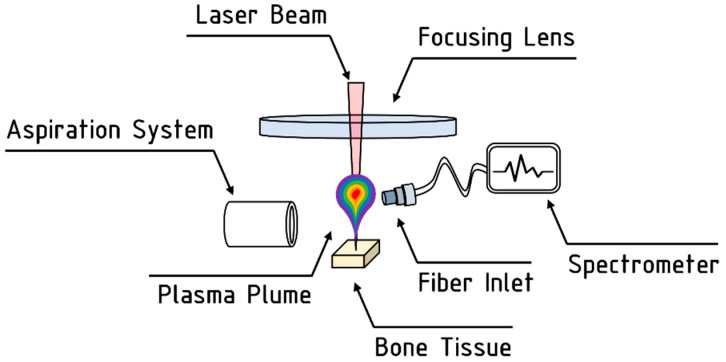
Schematic representation of the LIBS setup employed for the analyses.

LIBS technique was used to analyze the evolution of the elemental composition of the bone tissue during laser processing [24,25]. A spectrometer Ocean Optics HR4000CG-UV-NIR (Ostfildern, Germany—acquisition range of 200 nm–1100 nm; optical resolution < 0.1 nm), was employed to record emission spectra of the plasma plume emitted from the irradiated bone tissue. Before the start of the laser processing, the acquisition fiber was aligned to maximize the emission spectra, and its real-time acquisition was recorded by the OceanView software. A scheme of the LIBS setup is shown in Figure 4.

### 2.5. Processing Environment for Bone Tissue Cooling

Before post-processing characterization, all laser-processed samples were firstly dehydrated by multi-step baths in ethanol solutions (70% ethanol at 4 °C for 24 h, 90% ethanol at 4 °C for 1 h, 100% ethanol at 4 °C for 1 h). Three different characterization techniques were carried out on all processed samples:

- Confocal microscope (Zeiss SmartProof 5—Wetzlar, Germany) analyses were carried out to evaluate the ablation depth. To visualize the entire ablation cavity profile, several images were acquired and automatically stitched together by the analysis software (ZEN 2.3). Four measurements were carried on each ablation cavity in order to have statistically valid values and compensate for possible ablation inhomogeneities on the bottom of the cavities. For all ablated samples, the ablation rate was calculated as the ratio between the ablated volume (mm^3^) and the processing time (s).
- EDX (Bruker Quantax, Billerica, MA, USA) measurements were taken for all laser-processed samples in order to evaluate the laser-induced variation in atomic% of the main bone tissue components, such as C, O, Ca, Mg and P. To account for the semi-quantitative nature of the EDX analyses, all measured values were normalized with respect to the refence value of the same element measured on a non-laser processed area of the same sample. For each ablated cavity, three measurements were taken for statistical purposes. The ratio C^BEFORE^/C^AFTER^ between the atomic% of carbon C before and after the laser process was calculated and a threshold of C^BEFORE^/C^AFTER^ = 5 was defined to discriminate a healthy tissue from a calcified one for all samples. A more detailed description of this characterization approach is reported in [20].

**Figure 5:**
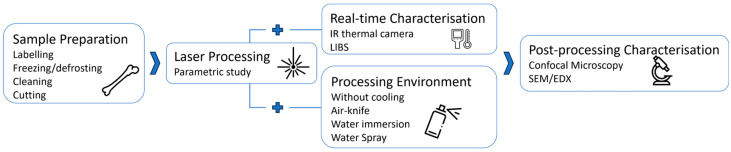
Workflow of the different steps implemented in this work.

Figure 5 summarizes the workflow of the experiments.

## 3. Results

For the sake of clarity, results on the evolution of the mean temperature (defined as the average maximal temperature recorded on the bone tissue surface at each laser passage and on each sample treated with the same set of laser process parameters) of bone tissue during fs laser processing are presented here in three parts. The first part (Section 3.1) considers the effect of different cooling environments on temperature and ablation rate for the same set of processing parameters (wavelength of 515 nm, P = 6.27 W, RR = 250 kHz, v = 2500 mm/s, h = 10 µm). The second part (Section 3.2) presents the evolution of the mean temperature with laser-induced thermal accumulation generated by varying the spatial overlap between successive impinging laser pulses. The processing parameters selected for the ablation tests were chosen according to previous results [20], where the maximum ablation rate was measured for processing at wavelength of 515 nm, repetition rate of 250 kHz and average power of 6.27 W. The last part (Section 3.3) shows the evolution of LIBS spectra with varying processing parameters and thus with the mean temperature of the laser processed bone tissue. Processing parameters employed for the tests are summarized in Table 2.

### 3.1. Effect of Processing Environment on Temperature, Ablation Rate and Calcination of Bone Tissue

**Figure 6:**
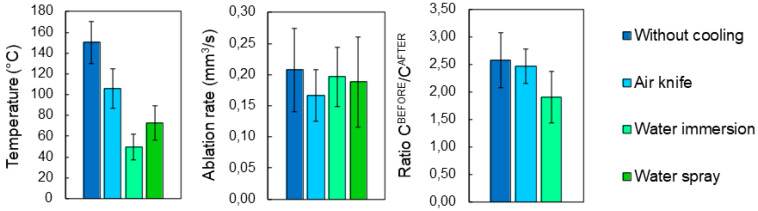
Evolution of (**left-hand side**) the mean temperature, (**center**) ablation rate and (**right-hand side**) ratio C^BEFORE^/C^AFTER^ as a function of cooling environment. For each processing environment, 5 cavities were ablated with a wavelength of 515 nm at *P* = 6.27 W, *RR* = 250 kHz and scanning speed *v* = 2500 mm/s. Error bars represent data standard deviations.

**Figure 7:**
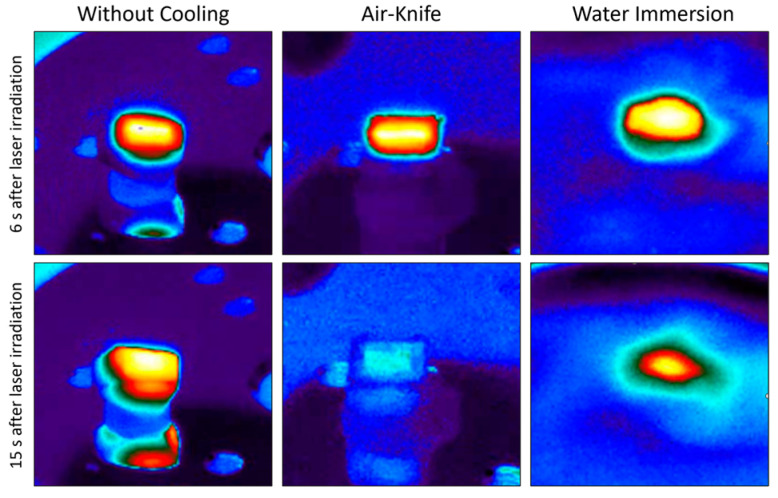
Images recorded from IR thermal camera 6 s and 15 s after the start of laser processing in three different processing environments (**left**: without cooling; **center**: air-knife; **right**: water immersion).

Figure 6 presents the evolution of mean temperature, ablation rate and calcination state of bone tissue treated with the same set of processing parameters by employing the four different cooling techniques previously introduced. The important impact of the cooling environment on the temperature reached by the bone tissue surface during laser processing is easily noticeable. Without any cooling system the temperature of the sample easily exceeds 150 °C. The use of an air knife already reduces the mean temperature to around 105 °C. However, water immersion allows much lower surface temperatures to be reached, with a mean temperature of about 49.5 °C, which is approximately the temperature threshold for bone tissue protein denaturation [22,23]. The use of air-and-water intermittent jet spray allows an intermediate temperature to be reached, with respect to that of a fully immersed sample with an average temperature of 72.8 °C. With these optimized processing parameters, and accounting for a non-negligible data variability, the ablation rate is not strongly affected by the specific cooling environment. Finally, no calcination is obtained: the carbon ratio (C^BEFORE^/C^AFTER^) is well below the calcination threshold of 5 identified in [20] and slightly decreases in water cooling environment. Figure 7 presents IR thermal camera images captured during laser processing in different environments at different times after the start of the laser processing. It is possible to observe the efficiency of both air and cooling environment, especially the improvement of heat diffusion in water environment. 

### 3.2. Effect of Laser-Induced Thermal Accumulation on Ablation Rate

**Figure 8:**
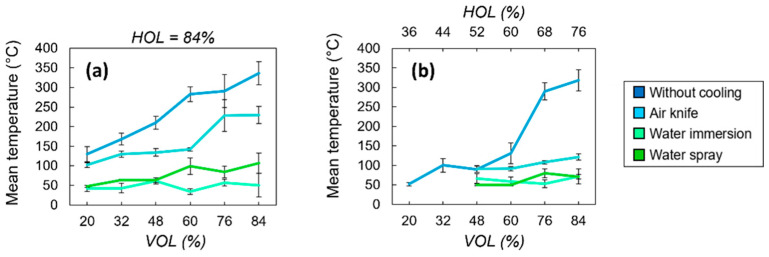
Evolution of the mean temperature as a function of the spatial overlaps *VOL* (**a**) and *HOL* (**b**) between successive laser pulses for the four different cooling methods. All cavities were ablated with a wavelength of 515 nm at *P* = 6.27 W and *RR* = 250 kHz. Error bars represent data standard deviations.

The evolution of the bone tissue mean temperature with the spatial overlap between successive laser pulses for the four different processing environments is shown in Figure 8. For a fixed spatial overlap between successive laser pulses of 84% in one scanning direction (Figure 8a), results show that the thermal accumulation linked to low thermal diffusivity of the bone tissue plays a key role in controlling the surface temperature during the laser processing. Indeed, in the case of the processing condition without any active cooling system in place, surface temperatures rise to about 350 °C. In this range of temperatures, the complete denaturation and carbonization of the tissue takes place and changes in the crystallography of the hydroxyapatite could already occur. This temperature decreases to 225 °C with air-knife cooling, to 100 °C with water vaporization on the sample surface and to 50 °C when the sample is completely submerged under a layer of water, regardless of the spatial pulse overlap. In the case of air-knife cooling, the temperature always stays above 100 °C and reaches values above 300 °C for high spatial overlaps between pulses, while in the case of water-cooling systems, the temperature stays below 100 °C and varies slowly with the spatial overlap. The decrease in surface temperature during laser processing is the most effective when thermal accumulation is reduced isotropically in both laser processing directions simultaneously (Figure 8b). The employment of an efficient water-based cooling system is mandatory when using processing parameters which lead to high thermal accumulation (*HOL*, *VOL* > 60%).

In the case of low spatial overlaps (*VOL*, *HOL* < 60%), the heat accumulation is intrinsically reduced due to the spreading of the laser-deposited energy over larger areas and thus bone tissue temperatures remain in a lower range regardless of the cooling environment. Therefore, an air-based cooling system could be sufficient to avoid the complete denaturation of the bone tissue. 

**Figure 9:**
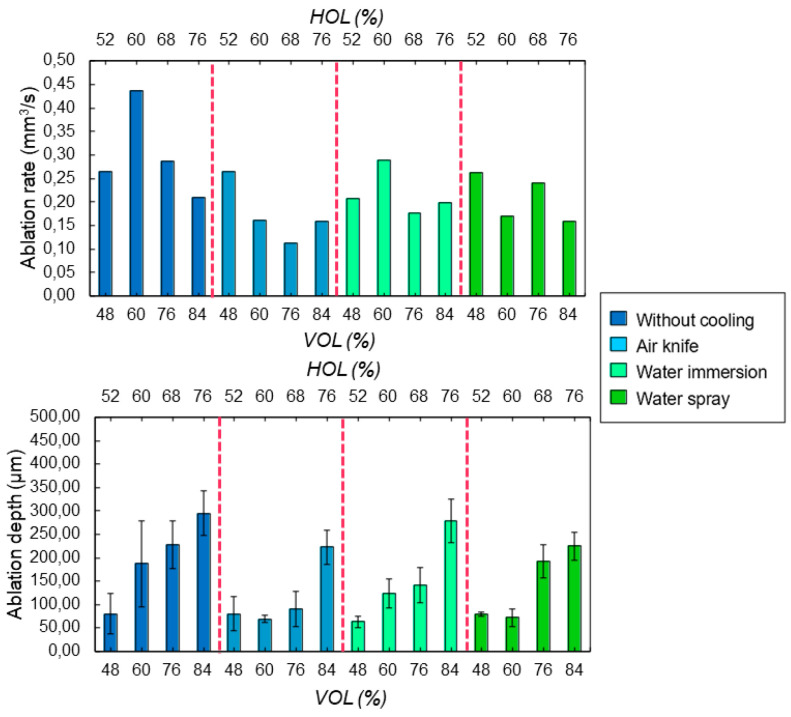
Evolution of (**top**) the ablation rate and (**bottom**) ablation depth as a function of the spatial overlaps *HOL* and *VOL* between successive laser pulses for the four different cooling methods. All cavities were ablated with a wavelength of 515 nm at *P* = 6.27 W and *RR* = 250 kHz.

Regardless of the heat accumulation, it is also important to guarantee a sufficient ablation rate during laser processing in a specific cooling environment, as well as the possibility to precisely control the ablation depths. Figure 9 presents the evolution of the ablation rate and ablation depths with respect to a variation of laser-induced thermal accumulation, i.e., of *HOL* and *VOL*. As already observed in a previous study [20], there is a threshold value of cumulative energy absorbed by the bone tissue, beyond which the ablation rate eventually drops. The same behavior is observed here as shown in Figure 9: optimum values of *HOL* and *VOL* exist in the case of cooling with water immersion or no cooling. A less important effect of the accumulated absorbed energy on the ablation rate values is noticeable for the case of water cooling. For the lowest spatial overlaps between successive laser pulses (*HOL*, *VOL* < 60%), where the temperature of the bone tissue does not exceed the temperature for protein denaturation, the variations of ablation rate in different cooling environments are within a few percent only with respect to that found without any active cooling system. For a given amount of laser-induced thermal accumulation, the presence of a water-based cooling environment does not affect the ablation rate or the ablation depth in a considerable way with respect to a cooling system based on pressured air.

### 3.3. LIBS Analyses

**Figure 10:**
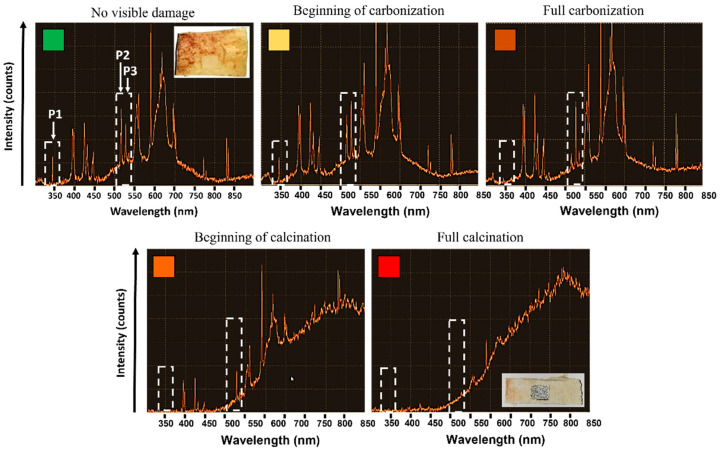
Evolution of the LIBS spectrum of bone tissue as a function of the laser-induced thermal state. Different states of the bone tissues are recognized are as follows: no visible damage (green, *N* = 4, *HOL* = 68%), beginning of carbonization (yellow, *N* = 4, *HOL* = 78%), full carbonization (light orange, *N* = 3, *HOL* = 81%), beginning of calcination (orange, *N* = 4, *HOL* = 81%) and full calcination (red, *N* = 4, *HOL* = 84%). All tests were carried at a wavelength of 1030 nm at *P* = 6.27 W, *RR* = 250 kHz and *VOL* = 84%. Dashed white rectangles highlight the three peaks (P1, P2, P3) which systematically change for different states of the bone tissue. Each section of the *Y*-axis corresponds to 200 intensity counts.

LIBS analyses were carried at different spatial overlap *HOL* and different number *N* of successive passes with a laser wavelength of 1030 nm to avoid the presence of artefacts in the emission spectra connected with the use of a visible laser wavelength. Visual observations of the state of the bone tissue were carried to identify and characterize the correspondent recorded emission spectra. Each test was repeated three times for sake of repeatability. The systematic variation of the intensity of three specific emission peaks was observed as the laser-induced thermal accumulation on the tissue varied. Figure 10 shows the appearance of the emission spectra for different states of the bone tissue, where the peaks P1@343.7 nm, P2@516.7 nm and P3@527.3 nm are highlighted by white arrows. Laser processing parameters are specified in the caption for Figure 7. Up to a temperature of about 150 °C, no changes in the spectra, as well in the visual appearance of the bone tissue, can be observed. Nonetheless, at these levels of temperature, protein and collagen denaturation are likely to have already occurred. As soon as the bone tissue begins carbonizing, P1 intensity rapidly decreases, while a simultaneous decrease and increase of P2 and P3 intensities occurs, respectively. This observation takes place systematically when the bone tissue enters a carbonization process. From this point forward, the intensity of P2 quickly drops and tends to zero once the calcination process starts. At the same time, and until calcination begins, P3 intensity maintains the same intensity. The beginning of the calcination process is characterized by the formation of a large shoulder in the emission spectrum and temperatures well above 350 °C. At this point, the three characteristic peaks are no longer visible.

**Figure 11:**
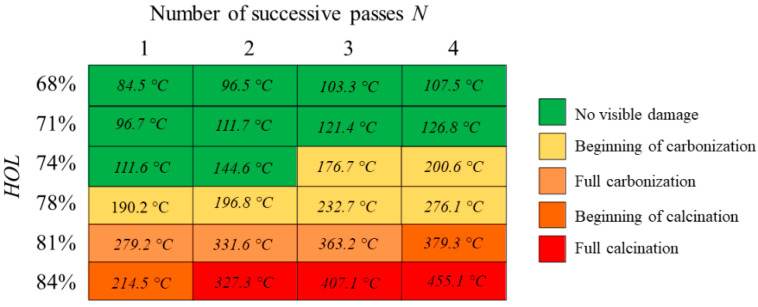
Values of maximum temperature recorded by FLIR camera on bone tissue samples as a function of the laser-induced thermal state. Different states of the bone tissues are recognized as follows: no visible damage (green, *N* = 4, *HOL* = 68%), beginning of carbonization (yellow, *N* = 4, *HOL* = 78%), full carbonization (light orange, *N* = 3, *HOL* = 81%), beginning of calcination (orange, *N* = 4, *HOL* = 81%) and full calcination (red, *N* = 4, *HOL* = 84%). All tests were carried at a wavelength of 1030 nm at *P* = 6.27 W, *RR* = 250 kHz and *VOL* = 84%.

To better understand the variations of the LIBS emission spectra with respect to process parameters, thermal imaging was carried simultaneously with the spectra acquisition. Figure 11 presents the mean values of the maximum temperature recorded by the FLIR camera during the laser processing at different spatial overlaps *HOL* and for each additional pass *N*. Laser processing parameters are specified in the caption of Figure 11. Given the thermal loads deposited by each successive pass, the higher the number of successive passes on the same irradiated cavity, the lower the temperature for carbonization and successive calcination. Ablating at increasing spatial overlap induces a slower temperature gradient that that observed by increasing the number of successive passes.

## 4. Discussion

Table 3 presents the highest values of ablation rate obtained in the four different cooling configurations and the corresponding mean temperature values reached during the laser interaction. As previously observed, laser processing in water environment led to slightly lower ablation rates, accounting for data variability on the measurements of the ablation depth values. In this configuration, the recorded mean temperature of the bone tissue is kept below 60 °C and the laser-induced damages are probably limited to a small degree of protein denaturation in the treated tissue. 

As shown in Table 4, the presence of a cooling environment allows for a decrease of the maximum peak temperature of the bone tissue during laser processing of almost 5 times that achieved via cooling by water immersion. While several methods have already been presented in the literature that show the benefits of a water-based processing environment, such as the vaporization of water on the sample or its total immersion under water bubbles [33,34,35,36,37], the results of this work demonstrate the uniqueness of fs laser processing to achieve ablation and cutting of bone tissue without the complete denaturation of the tissue itself, thanks to the possibility of ablating at competitive ablation rates at temperatures of about 50 °C. 

Although better results could likely be obtained with a continuous spray of high-pressure water, these results are very encouraging. This is because, as it is a water spray jet, its shows the possibility of achieving competitive results in terms of ablation rate at low mean temperature with very limited water consumption and with a clinical-environment-friendly setup. While such low temperature regimes can be obtained with the use of fs laser sources only, water sprays are indeed already used and are mandatory in many laser procedures to improve cutting quality, tissue regeneration and to increase ablation rates [38,39,40]. 

It is important to highlight that other intrinsic characteristics of bone tissue, i.e., its overall status before laser processing, must be taken into account while analyzing the effects of laser-induced thermal accumulation. The thermal properties of bone tissue depend on both its macroscopic and microscopic structures, as well as on its lipid and water contents [28]. These characteristics can strongly influence the rate of ablation as well as the thermal damage to the bone tissue during laser processing. Though the thermal conductivity could be considered relatively constant over the entire length of a femur, its value can be 50% higher for fresh bone than for dry bone; the hydration of the bone tissue directly influences the quality of ablation and the temperature during laser interaction [28]. 

This study also demonstrates that the combination of two inline contactless monitoring systems, such as the FLIR thermal camera and the LIBS technique, allows for a correlation of the surface temperature and the molecular state of the bone tissue in real-time directly during laser processing. However, once the tissue starts carbonizing, a blackening process of the bone tissue surface takes place, modifying its optical properties and its emissivity value [41]. The same happens for the calcination phenomenon, when the surface becomes rather greyish and less reflective. Inline monitoring results show that there is a link between the surface temperature and the plasma emission spectrum. If the mean temperature is lower than about 160 °C, the LIBS spectrum has the characteristic shape of a plasma spectrum of an intact bone tissue, without any signs of charring, and it is comparable to emission spectra found in the literature [42]. However, above this temperature, the first signs of carbonization are observed, and a systematic variation of three characteristic peaks, located at P1 = 343.7 nm, P2 = 516.7 nm and P3 = 527.3 nm, becomes visible. A comparative analysis reveals that these peaks could represent three molecules that together constitute an important part of the cortical bone [43,44,45], containing elements such as Na, Mg and Ca. By following the evolution of the first two peaks, P1 and P2, it is possible to observe the gradual disappearance of Na and Mg compounds with the increasing bone tissue temperature. The Ca-related peak (P3) increases its intensity before disappearing when full calcination is reached, highlighting a possible calcification mechanism taking place during the heating of the tissue. The first calcination state occurs at a surface temperature of 379.3 °C, when the emission spectra does not show any characteristic peaks. While LIBS is still an evolving method [46,47], it has already proven to be an essential technique in many fields of biology [48,49,50] and not only for the study of bone tissue: LIBS might have a high enough resolution to identify the presence of specific bacteria [51], as well as cancer cells, by observing the intensity ratio variation of certain molecules between healthy and cancerous tissue [52].

## 5. Conclusions

In this work, industrial-grade fs laser sources running in the visible (515 nm) and IR (1030 nm) regimes were employed to ablate cavities on porcine femurs. The aim of this study was to specifically analyze and characterize the thermal behavior of porcine femur undergoing fs laser ablation in order to be able to control the laser-induced damage and further optimize the laser processing of bone tissue with respect to previous works [20]. Results show that, by implementing a water-based processing environment, it is possible to achieve a larger process window at a higher average power for bone tissue ablation in which the bone tissue temperature remains within the protein denaturation temperature threshold with respect to the results obtained in [20]. This is a key outcome towards a clinical exploitation of the presented technology, for which higher process throughputs are necessary. Thermal imaging results collected by FLIR camera show that both competitive ablation rates of bone tissue and bone tissue friendly conditions with surface temperatures as low as 50 °C, are achievable. These low damage temperatures, which correspond to the very beginning of tissue protein denaturation, were achieved by using fs laser sources in a water-based cooling environment. The prospect of working at safe temperatures for the bone tissue is a unique advantage of employing fs laser systems and pushes the exploitation of this technology into surgical applications, where high-resolution ablation and the preservation of healthy tissue is crucial. Finally, real-time monitoring of molecular composition by LIBS techniques has been demonstrated to be a powerful tool for controlling the quality of laser ablation as well as the characteristics of ablated tissue, allowing the possibility to discriminate, in real time, between healthy and non-healthy tissue. Both the water spray cooling system and the LIBS analyses can be eventually easily implemented in robotic systems by employing fiber-guided fs laser systems.

## Figures and Tables

**Table 1:** Procedural parameters for pre-processing preparation of tissue samples.

	Femur Pre-Processing Procedural Parameters
Type of bone tissue	Porcine femur (age 7, male)/diaphysis
Storage conditions	−6 °C/1 d
Defrosting	RT/1 h/laminar flow system
Pre-cleaning	DI + ethanol @70%
Drying	RT/1 h/laminar flow system
Cutting	Diamond blade

**Table 2:** Laser processing parameters for all tests.

	Ablation Tests Visible Regime (515 nm)	LIBS Analyses IR Regime (1030 nm)
Laser source	Tangerine	Satsuma HP3
Average power *P* (W)	6.27	6.27
Repetition rate *RR* (kHz)	250	250
Number of successive passes *N*	5	1–4
Scanning speed *v* (mm/s)	1000–4000	1000–2500
Horizontal overlap *HOL* (%)	76; 68; 60; 52; 44; 36	68; 71; 74; 78; 81; 84
Interline distance *h* (μm)	4–20	4
Vertical overlap *VOL* (%)	84; 76; 60; 48; 32; 20	84

**Table 3:** Highest ablation rate values obtained by laser processing at 515 nm for each cooling method and its related laser processing parameters. *P* = laser average power (W), *RR* = laser repetition rate (Hz), *HOL*% and *VOL*% = spatial horizontal and vertical overlap between successive laser pulses, respectively.

Cooling Condition	*P* (W)	*RR* (kHz)	*HOL* (%)	*VOL* (%)	Max. Ablation Rate (mm^3^/s)	Mean Temperature (°C)
Without cooling	6.27	250	60	60	0.44	131 (SD 26 °C)
Air-knife			52	48	0.27	98.4 (SD 8 °C)
Water immersion			60	60	0.29	58.9 (SD 12 °C)
Water spray			52	48	0.26	50.3 (SD 2 °C)

**Table 4:** Maximum temperature values obtained by laser processing at 515 nm for each cooling method and its related laser processing parameters.

Cooling Condition	*P* (W)	*RR* (kHz)	*HOL* (%)	*VOL* (%)	Ablation Rate (mm^3^/s)	Max. Temperature (°C)
Without cooling	6.27	250	84	84	0.21	336.2
Air knife			84	84	0.21	229.6
Water immersion			76	84	0.20	71.9
Water spray			84	84	0.21	106.8

## Data Availability

Data are contained within the article.
